# Predictability of rotational tooth movement with orthodontic aligners comparing software-based and achieved data: A systematic review and meta-analysis of observational studies

**DOI:** 10.1177/14653125211027266

**Published:** 2021-06-27

**Authors:** Despina Koletsi, Anna Iliadi, Theodore Eliades

**Affiliations:** 1 Clinic of Orthodontics and Pediatric Dentistry, Center of Dental Medicine, University of Zurich, Zurich, Switzerland; 2 Department of Dental Biomaterials, School of Dentistry, National and Kapodistrian University of Athens, Attica, Greece

**Keywords:** aligner, prediction, Invisalign, tooth movement, rotation, systematic review, meta-analysis

## Abstract

**Objective:**

To evaluate all available evidence on the prediction of rotational tooth movements with aligners.

**Data sources:**

Seven databases of published and unpublished literature were searched up to 4 August 2020 for eligible studies.

**Data selection:**

Studies were deemed eligible if they included evaluation of rotational tooth movement with any type of aligner, through the comparison of software-based and actually achieved data after patient treatment.

**Data extraction and data synthesis:**

Data extraction was done independently and in duplicate and risk of bias assessment was performed with the use of the QUADAS-2 tool. Random effects meta-analyses with effect sizes and their 95% confidence intervals (CIs) were performed and the quality of the evidence was assessed through GRADE.

**Results:**

Seven articles were included in the qualitative synthesis, of which three contributed to meta-analyses. Overall results revealed a non-accurate prediction of the outcome for the software-based data, irrespective of the use of attachments or interproximal enamel reduction (IPR). Maxillary canines demonstrated the lowest percentage accuracy for rotational tooth movement (three studies: effect size = 47.9%; 95% CI = 27.2–69.5; *P* < 0.001), although high levels of heterogeneity were identified (I^2^: 86.9%; *P* < 0.001). Contrary, mandibular incisors presented the highest percentage accuracy for predicted rotational movement (two studies: effect size = 70.7%; 95% CI = 58.9–82.5; *P* < 0.001; I^2^: 0.0%; *P* = 0.48). Risk of bias was unclear to low overall, while quality of the evidence ranged from low to moderate.

**Conclusion:**

Allowing for all identified caveats, prediction of rotational tooth movements with aligner treatment does not appear accurate, especially for canines. Careful selection of patients and malocclusions for aligner treatment decisions remain challenging.

## Introduction

The advent of aligner treatment in orthodontic clinical practice dates back to the late 1990s, with the introduction of two different set-up aligner systems, grounded on the level and amount of displacement to be achieved (Boyd et al., 2000; Kim and Echarri, 2007). Interestingly, both targeted the satisfaction of the adult patients’ treatment need. In addition, perspectives on efficacy, aesthetics and comfort in a rapidly moving field in terms of technology advancements were considered. Since then, the vastly growing industry of aligner technologies, supported or led by the demands of the patients/clinicians and stakeholders, has gained increasing popularity. In this respect, patients treated with aligners in everyday orthodontic practice has been doubled in the United States, within a period of 6–7 years (Keim et al., 2014).

Lately, there has been an increasing interest with regard to treatment outcomes related to aligner therapy (Papageorgiou et al., 2020a, 2020b), forces and moments exerted to achieve tooth movement (Iliadi et al., 2019) and safety considerations (Iliadi et al., 2020). This has led to a number of studies aiming to systematically appraise the existing evidence within the field. Current evidence has suggested poorer treatment outcomes after orthodontic treatment with aligners in adults, compared to the gold standard of fixed appliances; however, this is considered low to moderate evidence quality, while the need for further well-designed and reported research remains solid (Papageorgiou et al., 2020a; 2020b).

Compromised treatment outcomes after use of aligners might be related to the inherent inability of the appliances to reach the amount of tooth movement anticipated at the beginning of the treatment, this being prescheduled through prediction models or company-driven prediction software (Chisari et al., 2014; Simon et al., 2014). Specific tooth movements have been identified as most prone to failure to achieve the anticipated predicted increment in practice. In essence, this is also related to tooth type and direction of movement (Charalampakis et al., 2018; Dai et al., 2019). Rotational movements have been reported to demonstrate the highest levels of inaccuracy in determining the prediction of the tooth change in position, with maxillary canines being the most affected teeth. According to some reports, canines demonstrate a mean rotational discrepancy between predicted and finally achieved movement of approximately 3.8°. This is important, since inaccurate prediction of tooth movements might be associated with prolonged duration of aligner treatment with an additional need for refinement strategies. Patient burnout and, most likely, increased potential for relapse tendency are further concerns (Papagiannis et al., 2021; Vagdouti et al., 2019; Vagdouti and Koletsi, 2020).

In view of the above, the aim of the present study was to review systematically the scientific evidence on the prediction potential of aligner software programs for rotational orthodontic tooth movements with the use of aligners. The null hypothesis was that there was no difference between predicted tooth movement and that achieved at the end of treatment.

## Methodology

### Protocol

The protocol was registered to the Open Science Framework (https://osf.io/cu4yz/) (Koletsi et al., 2020). The reporting scheme of the review was allied to the PRISMA statement (Liberati et al., 2009) and PRISMA statement for diagnostic test accuracy studies (McInnes et al., 2018).

### Eligibility criteria

#### Study design

Clinical (in vivo) studies referred to the predictability and accuracy of prediction of tooth movement. Eligible studies were observational designs, retrospective or prospective cohorts, and cross-sectional or case-control studies. In addition, randomised controlled trials were considered if these included a diagnostic accuracy section and at least one treatment group with aligners.

#### Population/type of tooth

There were no age or gender restrictions. Patients undergoing aligner orthodontic treatment were included. Any type of tooth with rotational movement plan was considered in maxillary and/or mandibular arch. Use of any adjuncts such as attachments or interproximal enamel reduction (IPR) strategies was also included.

#### Index tests

Index tests included virtual treatment plan and tooth movement, ClinCheck (Align Technology) for prediction of tooth movement and other prediction software models.

#### Target condition

Any study with achieved final orthodontic tooth movement (rotation), measured in casts (conventional or digital), was included.

#### Exclusion criteria

Studies not reporting on specific diagnostic methods for accuracy of tooth movement prediction related to rotation and other than in vivo studies were excluded. Studies not reporting on rotational movements were also excluded.

### Search strategy

Initially, an electronic search of seven databases was conducted up to 4 August 2020. This was supplemented by a hand search of the included studies for additional relevant publications. The databases included the following: PubMed via Medline, Scopus, Cochrane Central Register of Controlled Trials (CENTRAL) and Cochrane Database of Systematic Reviews (CDSR). Unpublished literature was searched within Open Grey, the ClinicalTrials.gov (www.clinicaltrials.gov), the National Research Register (www.controlled-trials.com). Keywords and MeSH terms included: ‘aligner’, ‘Invisalign’, ‘predicted tooth movement’ and ‘tooth rotation’. Search strategy for PubMed is presented in Appendix 1.

### Data collection

Data extraction was conducted using pre-piloted standardised forms by two independently working reviewers, non-blinded to the study origin or author identity. Entries involved study design, sample size, reference and index condition, outcomes as well as any other study specific information or related comments. Inconsistencies were discussed among reviewers until a consensus is reached. A third reviewer was consulted if needed and as appropriate, to settle any persisting disagreements.

### Risk of bias in individual studies

The methodological quality of the studies was determined using the QUADAS-2 (Quality Assessment of Diagnostic Accuracy Studies-2) tool (Whiting et al., 2011). Four domains were considered to determine the risk of bias and level of concern according to the applicability of the studies:

1. Patient selection: studies with a non-random or non-consecutive sample of patients, were judged as high risk of bias concerning the patient selection.
2. Index test: when diagnostic methods were interpreted without knowledge of the results of the reference standard, the index test domain was classified as high risk.
3. Reference standard: when reference standards were performed without knowledge of the index test results, the domain reference standard was classified as low risk of bias. Moreover, studies that did not report a reference standard were a priori excluded.
4. Flow and timing: when the reference standard was not used on all patients, or if all samples were not included in the analysis, the flow and time domain was classified as high risk of bias. Furthermore, when a long period had elapsed between the index test and the reference standard, the flow and time domain was classified as high risk.

Concerns about the applicability of the studies were determined as follows:

1. Patient selection: studies implemented with the inclusion of only a small sample size (< 10 patients) were characterised as having a high concern regarding applicability.
2. Index test: when the index test implementation was different from the review question, a high concern was documented for the specific study.
3. Reference standard: studies assessing the validation of the target condition by cast analysis and reliability assessment received a score of low concern regarding applicability.

### Summary measures and data synthesis

A qualitative as well as a quantitative analysis of the study outcomes was performed.

Quantitative syntheses of the studies’ findings were performed after exploring heterogeneity levels across individual reports. Random effects meta-analyses were conducted in view of the potential heterogeneity anticipated. Summary estimates for efficacy of prediction or prediction estimate with appropriate confidence bounds were presented for all applicable comparisons. The estimates were based on a percentage accuracy presentation based on ‘achieved’ against ‘predicted’ rotational tooth movement. Pooled estimates were ultimately presented if two or more studies were deemed eligible for a single comparison. Study authors were contacted for data request when information was missing within the published report.

### Additional analyses

Meta-regression analytical techniques were performed for the assessment of the effect of tooth on the pooled estimate (Monte-Carlo permutation test). In addition, sensitivity analyses were planned, with the exclusion of high risk of bias studies from the syntheses, if both high and lower risk of bias studies were finally included, in order to isolate and explore the effect of high risk of bias studies. Publication bias was planned to be examined through standard funnel plots if more than 10 studies were included in the quantitative syntheses.

### Quality of the evidence

The Grading of Recommendations Assessment, Development and Evaluation (GRADE) was used to assess the overall quality of the evidence stemming from the index/reference conditions and outcomes for evaluation (Balshem et al., 2011; Guyatt et al., 2008). According to GRADE, the overall body of evidence is rated as high, moderate, low and very low. Assessment of the body of evidence primarily involves assessment of study design. Assessment is made on the following domains: risk of bias; inconsistency; indirectness; imprecision; and publication bias. For the first four domains, the quality of evidence may be downgraded on the basis of either ‘serious’ or ‘very serious’ risks (1 or 2 levels, respectively); publication bias may either be suspected or undetected. For non-randomised/observational designs specifically, which theoretically start from a ‘low’ level of evidence, the perspectives for upgrade are as follows: a large or very large effect; a plausible residual confounding that may alter the effect; or a dose-response gradient. The level of evidence may be upgraded by 1 or 2 levels (large effect) or 1 level (plausible confounding, dose-response gradient).

## Results

### Search details

**Figure 1.**
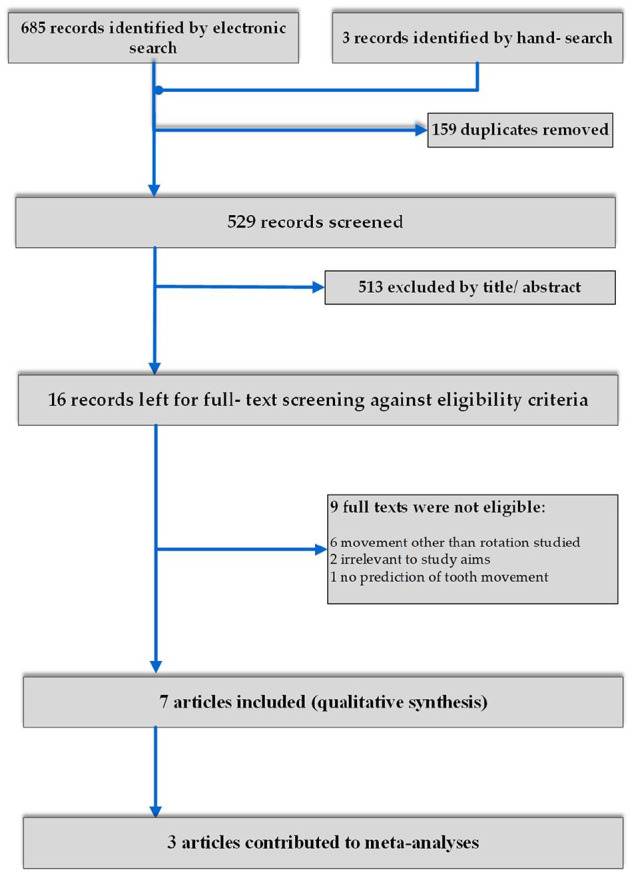
PRISMA flow diagram of study selection.

Study selection process, breakdown and number of included articles in qualitative and quantitative synthesis are presented in Figure 1. From an initial total of 529 unique records after duplicate removal, 16 articles passed to the full-text screening process, with seven ultimately included in the qualitative synthesis and three in the quantitative synthesis. The reasons for the exclusion of articles are outlined in Figure 1.

### Study design and characteristics

Detailed characteristics of included studies are presented in Table 1.

**Table 1.** Characteristics of included studies.

Study	Sample	Population/type of tooth	Index test	Target condition/outcome	Adjuncts	Comments
Charalampakis (2018) Retrospective cohort	20 Class I patients; 398 teeth, both arches, mostly mild crowding	3 men, 17 women; mean age 36.5 years; range 18.2–79.9 years; canines, premolars and incisors	ClinCheck®	Rotation following anterior aligner treatment, before refinement (Also: horizontal/vertical displacement, transverse changes)	No restriction on attachment use/according to doctor’s prescription	Anterior Invisalign® (SmartTrack material); superimpositions made on virtual software based on stationary posterior teeth
Grunheid (2017) Retrospective cohort	30 patients; both arches, ~600 teeth mostly mild crowding	13 men, 17 women; mean age 21.6± 9.8 years; all teeth	ClinCheck®	Rotation, following Invisalign treatment (also: tipping, torque, translation)	Attachment use and IPR as prescribed	Invisalign®; superimpositions based on best-fit surface-based registration
Haouili (2020) Prospective cohort	38 patients; both arches, 899 teeth	13 men, 25 women; included teenage patients; mean age 36 years; all teeth	ClinCheck®	Rotation, following Invisalign treatment (also: tipping, intrusion, extrusion)	Both arches average 6 attachments and <1 mm IPR	Invisalign®; superimpositions based on best-fit surface-based registration
Kravitz (2008) Prospective cohort	31 patients (53 canines: 33 maxillary, 20 mandibular); anterior crowding <5 mm	13 men, 18 women; age > 18 years (mean age 29.4 years); canines. Part of a larger clinical study	ClinCheck®	Rotation, following anterior aligner treatment	3 groups: attachments only, IPR only, no attachments or IPR	Anterior Invisalign®; superimpositions made on virtual software based on stationary posterior teeth
Kravitz (2009) Prospective cohort	37 patients (401 maxillary and mandibular incisors and canines); anterior crowding <5 mm	14 men, 23 women; mean age 31 years; canines and incisors	ClinCheck®	Rotation, following anterior aligner treatment (also: expansion, constriction, intrusion, extrusion, tipping)	No restriction on attachment use and IPR; use of IPR in 45% of sample, use of attachments in 17% according to doctor’s prescription	Anterior Invisalign®; superimpositions made on virtual software based on stationary posterior teeth
Lombardo (2017) Retrospective cohort	16 patients (345 maxillary and mandibular teeth); crowding <5 mm	6 men, 10 women; mean age 28.6 years; all teeth	VAM software (Vectra, Canfield Scientific, Fairfield, NJ, USA)	Rotation following F22 aligner treatment (also: mesiodistal tipping, vestibulolingual tipping)	No restriction on attachment use and IPR according to doctor’s prescription	F22 aligners; superimpositions based on best-fit 100-reference point registration
Simon (2014) Retrospective cohort	30 patients (49 teeth, maxillary molars and incisors; maxillary and mandibular premolars)	11 men, 19 women; included teenage patients; mean age 32.9 ±16.3 years; central incisors, premolars, molars	ClinCheck®	Rotation of premolars following Invisalign treatment (also: molar distalisation, central incisor torque)	Subgroups with ± attachment use	Invisalign®; superimpositions using a surface matching algorithm

IPR, interproximal reduction.

Of the seven studies included in the review (Charalampakis et al., 2018; Grunheid et al., 2017; Haouili et al., 2020; Kravitz et al., 2008, 2009; Lombardo et al., 2017; Simon et al., 2014), three were prospective cohorts (Kravitz et al., 2008, 2009; Haouili et al., 2020), while the remaining four were retrospective cohorts (Charalampakis et al., 2018; Grunheid et al., 2017; Lombardo et al., 2017; Simon et al., 2014). One study (Kravitz et al., 2008) was based on a larger clinical study with the entire sample presented in the report of Kravitz et al. (2009). However, it was included separately in the review, as the groups identified were distinct in nature; in addition, this did not contribute twice to the meta-analyses performed. Sample sizes for the studies included in the review were in the range of 20–38 within eligible studies. The number of teeth included in studies was in the range of 49–899. The range of mean ages of patients comprising the studies’ samples was 21.6–36.5, and only two studies specifically reported inclusion of adolescent patients within their sample (Haouili et al., 2020; Simon et al., 2014). Six of the seven studies included patients treated with Invisalign® (Align Technology, Santa Clara, CA, USA) and one (Lombardo et al., 2014) used F22 Aligners (Sweden & Martina, Due Carrare, Italy). The VAM software (Vectra, Canfield Scientific, Fairfield, NJ, USA) was the software described and used in the latest report for the prediction of tooth movement strategies, while the remaining studies used the ClinCheck proprietary software of Align Technology. Superimposition of models to test the differences between predicted and actually achieved tooth movements was made on posterior stationary or almost-stationary teeth (Charalampakis et al., 2018; Kravitz et al., 2008, 2009), best-fit surface or point-based registrations (Grunheid et al., 2017; Haouili et al., 2020; Lombardo et al., 2017) or surface matching algorithms (Simon et al., 2014). Use of attachments and/or IPR strategies were performed without restriction, based on the clinician’s treatment decision, in four studies (Charalampakis et al., 2018; Grunheid et al., 2017; Kravitz et al., 2009; Lombardo et al., 2017). The rest of the studies included different subgroups of patients with or without attachments/IPR (Haouili et al., 2020; Kravitz et al., 2008; Simon et al., 2014) (Table 1).

### Risk of bias within studies

**Figure 2.**
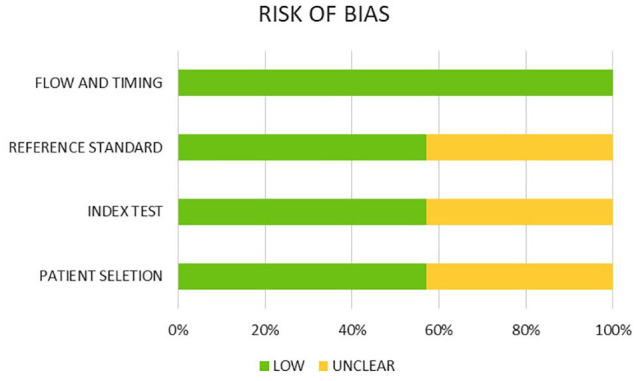
Risk of bias assessment of included studies (percentage frequency distribution per item), according to QUADAS-2 tool.

The overall risk of bias was rated as unclear in three out of seven studies (Charalampakis et al., 2018; Kravitz et al., 2008, 2009) and as low in four studies (Grunheid et al., 2017; Haouili et al., 2020; Lombardo et al., 2017; Simon et al., 2014). The main domains contributing to unclear risk of bias were patient selection, index test or reference standard. Specifically for patient selection, in the unclear risk of bias studies, this was due to non-random or non-consecutive selection, without description of patient recruitment details. For the latter two domains, the level of recording was based on adequate description of whether interpretation of the diagnostic methods was done blindly and independently, without prior knowledge of each other test (Table 2). The percentage distribution of the risk of bias across domain of the QUADAS-2 tool is presented in Figure 2.

**Table 2.** Risk of bias assessment and applicability concerns (QUADAS-2).

Study	Risk of bias				Applicability concerns		
	Patient selection	Index test	Reference standard	Flow and timing	Patient selection	Index test	Reference standard
Charalampakis (2018)	Unclear	Unclear	Unclear	Low	Low	Low	Low
Grunheid (2017)	Low	Low	Low	Low	Low	Low	Low
Haouili (2020)	Unclear	Low	Low	Low	Low	Low	Low
Kravitz (2008)	Low	Low	Low	Low	Low	Low	Low
Kravitz (2009)	Low	Low	Low	Low	Low	Low	Low
Lombardo (2017)	Unclear	Unclear	Unclear	Low	Low	Low	Low
Simon (2014)	Low	Unclear	Unclear	Low	Low	Low	Low

### Effects of interventions, meta-analyses and additional analyses

**Figure 3.**
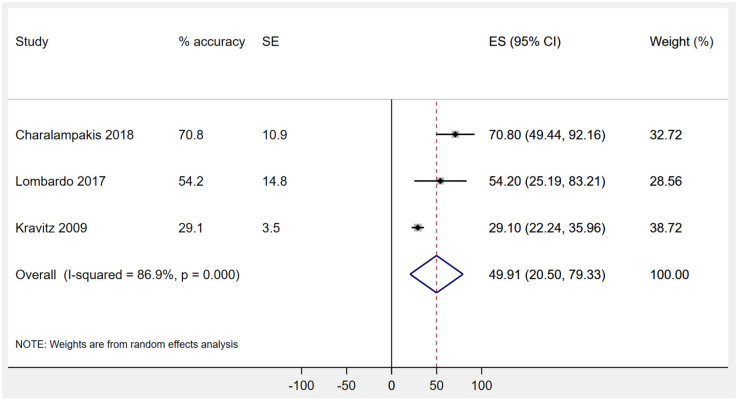
Random effects meta-analyses for the percentage accuracy of predicted rotational movement of the mandibular canine.

As previously noted, three studies contributed to meta-analyses or additional analyses (Charalampakis et al., 2018; Kravitz et al., 2009; Lombardo et al., 2017). The anterior teeth of maxillary and mandibular arch and premolars were analysed separately. Canine teeth showed the lowest percentage accuracy for prediction of rotational movement, with upper canines exhibiting an accuracy of 47.9% (three studies: 95% confidence interval [CI] = 27.2–69.5; *P* < 0.001) and lower canines exhibiting an accuracy of 49.9% (three studies: 95% CI = 20.5–79.3; *P* = 0.001) (Figure 3). In contrast, the percentage accuracy of predicted tooth movement for mandibular incisors (two studies: 70.7%; 95% CI = 58.9–82.5; *P* < 0.001) as well as mandibular premolars (two studies: 67.0%; 95% CI = 52.2–81.8; *P* < 0.001) appeared higher (Table 3).

**Table 3.** Results of meta-analyses, according to tooth type and arch.

Syntheses-rotation % accuracy	No. of studies	Effect size (%)	95% CI	*P* value	I^2^ (%)
Maxillary central incisors	2	54.5	47.6–61.4	<0.001	0.0
Maxillary lateral incisors	2	51.5	30.1–72.8	<0.001	65.4
Maxillary canines	3	47.9	27.2–69.5	<0.001	75.5
Maxillary premolars	2	64.4	44.9–84.0	<0.001	54.3
Mandibular incisors	2	70.7	58.9–82.5	<0.001	0.0
Mandibular canines	3	49.9	20.5–79.3	0.001	86.9
Mandibular premolars	2	67.0	52.2–81.8	<0.001	0.0

CI, confidence interval.

**Figure 4.**
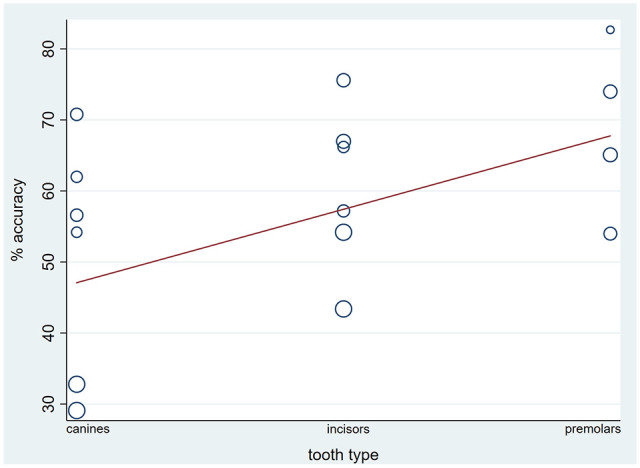
Bubble-plot for the effect of type of tooth on percentage accuracy, based on meta-regression analytical technique.

Meta-regression based on three studies and 16 tooth-pair comparisons overall revealed weak evidence of a significant effect of tooth type, irrespective of arch (Monte-Carlo permutation test, *P* = 0.04) (Figure 4).

Further sensitivity analyses were not implemented, and publication bias could not be assessed, due to the low number of studies included in the meta-analyses.

### Quality of the evidence

The quality of the existing evidence for the percentage accuracy of rotational tooth movements, between predicted and achieved movement, was rated as low to moderate overall. This pertained to anterior teeth and premolars of both maxillary and mandibular arch. A large observed effect was the most common reason for upgrade. High heterogeneity levels, denoting inconsistency, contributed to downgrading the quality of the evidence (Table 4). Apparently, this means that further research is likely or very likely to have an important impact on our confidence in the estimated effect.

**Table 4.** Quality of the evidence according to GRADE.

Outcomes (% accuracy per tooth type)	No. of teeth (studies) Follow-up	Quality of the evidence (GRADE)*	Relative effect (95% CI)
Maxillary central incisors	99 (2 studies)	⊕⊕⊕⊝ Moderate^†^ due to large effect	54.5 (47.6–61.4)
Maxillary lateral incisors	99 (2 studies)	⊕⊕⊝⊝ Low^†‡^ due to inconsistency, large effect	51.5 (30.1–72.8)
Maxillary canines	122 (3 studies)	⊕⊕⊝⊝ Low^†‡^ due to inconsistency, large effect	47.9 (27.2–69.5)
Maxillary premolars	108 (2 studies)	⊕⊕⊝⊝ Low^†‡^ due to inconsistency, large effect	64.4 (44.9–84.0)
Mandibular incisors	131 (2 studies)	⊕⊕⊕⊝ Moderate^†^ due to large effect	70.7 (58.9–82.5)
Mandibular canines	120 (3 studies)	⊕⊕⊝⊝ Low^†‡^ due to inconsistency, large effect	49.9 (20.5–79.3)
Mandibular premolars	115 (2 studies)	⊕⊕⊕⊝ Moderate^†^ due to large effect	67.0 (52.2–81.8)

* GRADE Working Group grades of evidence. High quality: Further research is very unlikely to change our confidence in the estimate of effect. Moderate quality: Further research is likely to have an important impact on our confidence in the estimate of effect and may change the estimate. Low quality: Further research is very likely to have an important impact on our confidence in the estimate of effect and is likely to change the estimate. Very low quality: We are very uncertain about the estimate.

† Large effect.

‡ High heterogeneity levels.

CI, confidence interval.

## Discussion

### Findings in context

The findings of this systematic review suggest an inaccurate prediction potential for rotational tooth movements through the use of the currently industry-available simulation programs; this might allow for speculations with regard to a diminished aligner efficacy for certain types of tooth movements. The null hypothesis was therefore rejected. Canine derotation strategies for prediction was the most afflicted type of tooth movement in terms of prediction accuracy and this finding was further supported through the meta-regression. The constraints with regard to rotational tooth movement with specific identification of canines, have been originally documented by the first clinical study in the field (Kravitz et al., 2008, 2009). Canine derotation has been identified as the second least accurate movement overall, following incisor extrusion, leaving about a 50% gap between predicted and achieved tooth movement after completion of the main active phase of the treatment. The respective figure for incisor extrusion has been found to correspond to about 28%.

Following advancements in technology, the biomedical field has gained in knowledge for the benefit and safety of the patients; oral health and orthodontics are surely representative examples of technological applications to practice (Eliades et al., 2020; Eliades and Zinelis, 2021; Mao, 2010). Aesthetic considerations and patients’ demands for ‘invisible’ orthodontic treatment have imposed certain goals for prediction of the anticipated tooth movement, with increased interest in tooth alignment (Ke et al., 2019; Robertson et al., 2020).

### Prior research and implications for practice

Research in the field of prognosis and accuracy of prediction of the desired tooth movement has resulted in a small but not insignificant group of primary studies, with the majority being conducted in the last 3–4 years (Charalampakis et al., 2018; Haouili et al., 2020; Lombardo et al., 2017). In-house simulation software has been developed and utilised by companies in an attempt to provide visualisation of treatment outcome, following a range of individually planned tooth movement increments (Elkholy et al., 2019; Krieger et al., 2012; Simon et al., 2014). In this respect, prediction of anticipated tooth movements is constantly used by the companies of aligner providers in order to estimate the sequential change of aligners during treatment. This may ultimately lead to a rough estimation of treatment duration, indeed conditional on the patients’ compliance. In addition, there is a high probability that the clinician may use this simulated illustration of treatment as a piece of information for the patients, who might probably be interested in a visual representation of the course and outcome of their treatment. The latter might potentially bear an impact on informed consent of patients to treatment. On the same grounds, acknowledgement of the potential limitations or drawbacks of the reported simulation programs by the clinician, is expected; this would help patients arrive at a more informed and evidence-based decision.

In this respect, a number of studies have been identified by the present systematic review and meta-analysis, all published within the last 12 years (Charalampakis et al., 2018; Grunheid et al., 2017; Haouili et al., 2020; Kravitz et al., 2008, 2009; Lombardo et al., 2017; Simon et al., 2014). Previous reports had emerged, albeit none included any attempt to mathematically synthesise available data (Kassam and Stoops, 2020; Robertson et al., 2020) in the field. In addition, previous reviews covered an utterly different aspect of research endeavour on aligners, mostly describing comparison with fixed appliances and treatment outcomes (Papageorgiou et al., 2020a, 2020b).

The efficacy potential of aligner treatment with regard to rotational movement increments of canines appear to lag behind to what is called a desirable effect, or to other tooth types and movements (Haouili et al., 2020); however, some improvements might be considered justified over the years. It might be likely that the anatomy and shape of the crown of the canines is a significant restricting parameter and this might hinder the aligners’ true potential for a more effective rotational movement; one might further argue that the curved anatomical surface of the canines could potentially reduce the dynamic of the attachment’s grip, if one is used in these teeth. In the same direction, interproximal contacts of rotated canines might also be considered a significant predictor for the diminished efficacy of tooth movement, especially in the absence of interproximal reduction of the enamel (IPR). However, the direction of derotation has been documented to play a role in the final outcome, with distal movement demonstrating less accuracy than mesial (Haouili et al., 2020). This finding is possibly allied to the actual contact area between canine and premolar, or further potential challenges of providing enamel reduction in this area. Breakdown and subgroup analyses based on the specific direction of rotational movements could not be determined, for any of the tooth groups examined, in view of the apparent scarcity of evidence from primary studies.

Studies included in the quantitative synthesis of this review described no restriction in the use of attachment grips or performance of interproximal enamel reduction strategies, thus most likely representing a real-clinical practice scenario. Although various types/shapes of attachment grips or practices of interproximal enamel reduction have been reported as potential prognostic factors for better efficacy of rotational tooth movement, this does not necessarily translate into an identified substantial effect in practice. The early study by Kravitz et al. (2008) exclusively assessed the net effect of attachment placement or IPR strategies on the accuracy of rotational canine movement with Invisalign®; the findings demonstrated null additional impact compared to aligner treatment without such adjuncts. These conclusions were corroborated by the latest study by Simon et al. (2014) on the efficacy of derotation of premolar teeth with or without attachment placement, raising concerns about the extensive use or prescription of such adjuncts; such practices have been additionally criticised for other patient-related conditions, pertaining to risk management considerations and safety in clinical settings (Eliades and Koletsi, 2020; Eliades et al., 2020). As such, careful selection of patient and malocclusion cases that may be successful candidates for the use of such types of adjuncts during aligner treatment should be critically implemented.

In addition, although the effect of the magnitude of rotational tooth movement could not be formally assessed due to the scarcity of the available evidence, sporadic reports identify an amount of rotation greater than 15° as a significant risk factor for decreased accuracy for rotational prediction; thus, potentially demonstrating a declining efficacy of the aligners to purely accomplish challenging treatment goals (Kravitz et al., 2009, Simon et al., 2014). It is notable that accuracy of rotational movements of more than 15° for canines and premolars may drop to percentages as low as 18.8% and 23.6%, respectively.

### Strengths and limitations

To our knowledge, this is the first meta-analysis on the comparison of specific types of predicted and actually achieved tooth movements (sp. rotation), after aligner treatment. A comprehensive and up-to-date search strategy to identify all eligible articles within published and unpublished literature was conducted, while the most rigorous guidelines for reporting and risk of bias assessment have been considered. The protocol for this study was a priori registered, thus eliminating the risk of selective reporting (Fleming et al., 2015; Koufatzidou et al., 2019).

Nevertheless, specific caveats do exist. The generalisability of the study findings is limited at present with any implication made being linked only to rotational type of tooth movement and to certain tooth groups. However, the reported data represent the current state of the evidence with regard to one of the most challenging and most documented types of tooth movement with aligners. Quantitative syntheses were based on a small number of studies, while in some cases statistical heterogeneity was evident (Koletsi et al., 2018). This might have imposed a bearing on the precision of the recorded estimates and the respective confidence bounds. Last, the most recent advancements in materials or adjuncts used in aligner treatment, might have not been reflected within the present SR report, due to the lack of relevant primary studies. In this respect, if additional new studies do emerge in the field, it might be deemed meaningful to consider an update of the existing data.

## Conclusion

According to available evidence on the comparison between software predicted and actual rotational tooth movement, the percentage accuracy was low for anterior teeth and premolars. The quality of the existing evidence did not substantiate strong confidence in the estimated and observed effect. Patients eligible for aligner treatment should be selected carefully and considerations of patients’ values and preferences should be prioritised. Efficacy of treatment outcome should be considered along with patient burnout and safety.

## Supplemental Material

sj-doc-1-joo-10.1177_14653125211027266 – Supplemental material for Predictability of rotational tooth movement with orthodontic aligners comparing software-based and achieved data: A systematic review and meta-analysis of observational studies Click here for additional data file. Supplemental material, sj-doc-1-joo-10.1177_14653125211027266 for Predictability of rotational tooth movement with orthodontic aligners comparing software-based and achieved data: A systematic review and meta-analysis of observational studies by Despina Koletsi, Anna Iliadi and Theodore Eliades in Journal of Orthodontics

## Appendix 1

PubMed via Medline

No filters applied

Date: 04.08.2020

1. orthodontic aligner
2. thermoplastic aligner
3. Invisalign
4. tooth aligner
5. clincheck
6. 1 OR 2 OR 3 OR 4 OR 5
7. tooth movement
8. predicted tooth movement
9. tooth rotation
10. rotational tooth movement
11. orthodontic movement
12. tipping movement
13. torque movement
14. 7 OR 8 OR 9 OR 10 OR 11 OR 12 OR 13
15. accuracy
16. prediction
17. efficiency
18. efficacy
19. 15 OR 16 OR 17 OR 18
20. 6 AND 14 AND 19
